# Nursing workforce trends in Kazakhstan, 2003–2024: an interrupted time series and joinpoint regression analysis in the context of educational reform

**DOI:** 10.3389/fpubh.2026.1913586

**Published:** 2026-09-28

**Authors:** Dinara Ospanova, Raushan Issayeva, Lyazzat Alibekova, Makhigul Maxudova, Neilya Ussebayeva, Meruyert Suleimenova

**Affiliations:** 1 Department of Nursing, Al-Farabi Kazakh National University, Almaty, Kazakhstan; 2 Higher Medical College of the Almaty Public Health Department, Almaty, Kazakhstan; 3 Department of Public Health, Asfendiyarov Kazakh National Medical University, Almaty, Kazakhstan

**Keywords:** Central Asia, educational reform, health workforce, interrupted time series analysis, joinpoint regression, Kazakhstan, nursing workforce

## Abstract

**Introduction:**

Kazakhstan introduced the Applied Bachelor of Nursing programme in 2014 as part of a broader effort to upgrade nursing education to degree level. Over the same period, the population grew from 14.9 to 20.0 million and the hospital network contracted by 27%. Whether these simultaneous pressures on workforce supply and demand translated into measurable changes in nursing density has not been examined.

**Methods:**

We conducted an ecological time series study using administrative data from the Bureau of National Statistics (2003 to 2024). The primary outcome was nursing and midwifery personnel per 10,000 population, a composite indicator of which nurses constitute approximately 77%. We combined interrupted time series analysis (ITSA) with a pre-specified 2014 intervention point and data-driven joinpoint regression to identify structural breakpoints. A generalized least squares (GLS) model with second-order autoregressive AR(2) errors served as the primary specification, supported by six sensitivity analyses, regional analysis, and autoregressive integrated moving average (ARIMA) forecasting to 2035.

**Results:**

Nursing density rose from 77.4 per 10,000 in 2003 to a peak of 101.2 in 2012, then stabilized between 93.3 and 100.0 through 2024. Joinpoint regression placed the structural breakpoint at approximately 2012 (95% CI: 2010 to 2014), 2 years before the reform. Sensitivity analyses confirmed this was not a denominator artifact. The GLS model identified significant coefficients at 2014 (level change: minus 5.45, *p* = 0.001; slope change: minus 2.16 per year, *p* < 0.001), but these reflected the proximity of the cut point to the actual trend reversal rather than a causal effect. The nurse-to-physician ratio declined from 2.62 in 2012 to 2.30 in 2024, below the WHO-recommended 3:1. Regional density ranged from 72.0 to 134.8 per 10,000 in 2024. The forecast projected 111.0 per 10,000 by 2035 (95% prediction interval: 92.6 to 129.4).

**Conclusions:**

The deceleration of nursing density growth preceded the 2014 reform by 2 years and coincided with the end of post-independence recovery and hospital contraction. Kazakhstan's density exceeds the WHO European Region average, but the nurse-to-physician ratio has been declining and educational output cannot keep pace with population growth. Expanding programme capacity and setting explicit ratio targets are needed.

## 1 Introduction

The global nursing workforce faces a projected shortage of 5.7 million nurses by 2030, concentrated in low-and middle-income countries (1). In response, many countries have reformed nursing education by upgrading vocational diploma programmes to university-level bachelor degrees, aiming to improve clinical competence and expand scope of practice (2, 3). Whether these educational reforms translate into measurable changes in workforce capacity at the population level remains unclear.

Kazakhstan, an upper-middle-income country with a population of 20 million, provides a particularly informative setting for studying this question. Following independence in 1991, the country lost nearly half its nursing workforce within a decade as the number of nurses declined from 180,000 to 92,000 due to economic crisis, low wages, and emigration (4, 5). Recovery began in the early 2000s alongside economic growth and increased government health expenditure (6). The healthcare system, structured on the Soviet Semashko model, trained mid-level medical personnel through technical and vocational colleges to perform clinical tasks under direct physician supervision (7, 8). The nurse-to-physician ratio remained near 2:1, well below the WHO-recommended minimum of 3:1 and far from the ratios of 3.0 to 4.7 observed in OECD countries with strong primary healthcare systems (9, 10).

Two major educational reforms reshaped nursing training during the study period. In 2007, Kazakhstan introduced university-level bachelor programmes in nursing for the first time. In 2014, the Ministry of Health formally initiated the Applied Bachelor of Nursing programme (Order No. 417), designed in partnership with international institutions to align nursing education with European Directive 2005/36/EC (11). This was followed by formal state educational standards in 2017, accelerated retraining tracks for working nurses, and a comprehensive Nursing Development Plan (11, 12). These reforms aimed to transform nurses from physician assistants into independent professionals capable of clinical assessment, care planning, and disease management (13). However, whether such reforms lead to expansion of the workforce, rather than improvements in quality alone, remains uncertain.

Previous research on Kazakhstan's health workforce has focused on rural workforce availability (14), rural healthcare infrastructure (15), epidemiological workforce trends (16), and regional inequality in resource distribution (17). Jobalayeva et al. (14) used ITSA with ARIMA models to evaluate a 2009 rural incentive scheme and found that per capita gains were modest. A sub-regional review by Dussault et al. found that the aggregate number of practicing nurses across Central Asia declined from 493,644 in 2010 to 398,704 in 2020, although Kazakhstan increased its absolute nursing workforce during this period (18). No study has examined the national nursing density trend in relation to the educational reform timeline. As a result, policymakers lack evidence on whether the reform effort has been sufficient to maintain workforce capacity in the context of rapid population growth.

ITSA is considered one of the strongest quasi-experimental designs for evaluating population-level policy interventions when randomized trials are not feasible (19, 20). The method estimates immediate level changes and slope changes in an outcome following an intervention, while controlling for the pre-existing trend (20, 21). ITSA has been used to evaluate health workforce policies in Kazakhstan (14, 16) and was applied by Semenova et al. (22) to examine medical education reforms and ophthalmology workforce trends in the same setting. Recommendations for adequate statistical power suggest a minimum of 8 to 12 data points on each side of the intervention (21, 23); our design, with 11 pre- and 11 post-intervention observations, meets these criteria.

The aim of this study was to examine trends in nursing workforce density in Kazakhstan from 2003 to 2024 and to test whether the 2014 initiation of the Applied Bachelor reform was associated with changes in the level or trajectory of nursing density. We combined ITSA to test the reform hypothesis with data-driven joinpoint regression to identify structural breakpoints without pre-specified assumptions, assessed regional variation, and projected nursing density to 2035. Understanding whether large-scale nursing education reforms translate into measurable workforce expansion is essential for countries facing simultaneous educational transition and rapid demographic growth.

## 2 Materials and methods

### 2.1 Study design

We conducted an ecological time series study using publicly available secondary data. We applied two complementary analytical approaches: ITSA to test a pre-specified reform hypothesis, and joinpoint regression to identify data-driven structural breakpoints without assumptions about intervention timing (19, 20, 24).

### 2.2 Setting

Kazakhstan is an upper-middle-income Central Asian republic whose population grew from 14.9 million in 2003 to 20.0 million in 2024. Nursing education historically relied on technical and vocational colleges. In 2007, universities introduced bachelor-level nursing programmes. In 2014, the Ministry of Health initiated the Applied Bachelor programme (Order No. 417). In 2017, formal state educational standards were approved (Order No. 471), and the programme expanded. A full reform timeline appears in Supplementary Table S1.

### 2.3 Data sources

We used three publicly available datasets from the Bureau of National Statistics of the Agency for Strategic Planning and Reforms of the Republic of Kazakhstan (stat.gov.kz): (1) number of mid-level medical personnel (indicator code 631502), which includes nurses, midwives and feldshers workers registered at year end; (2) number of physicians (indicator code 631501); and (3) population at the beginning of the period (indicator code 611101). The Bureau recalculated population data from 2009 onward following administrative boundary changes, which may have introduced a denominator shift affecting density estimates around that year. We retrieved regulatory documents from the legal information system Adilet (adilet.zan.kz). All datasets were accessed in March 2026.

### 2.4 Variables

The primary outcome was nursing workforce density, defined as the number of mid-level medical personnel per 10,000 population. This indicator is composite, comprising nurses, midwives and feldshers; according to the Ministry of Health, nurses constitute approximately 77% of this category (25). Secondary outcomes included absolute nursing personnel numbers, physician density per 10,000 population, and the nurse-to-physician ratio. For the ITSA, we constructed time (counter from 0 in 2003, incrementing by 1 each year), intervention (1 for years from 2014 onward, 0 otherwise), and time-after-intervention (years since 2014 multiplied by the intervention indicator, capturing slope change) variables (20, 21).

### 2.5 Statistical analysis

#### 2.5.1 Joinpoint regression

We applied segmented regression without pre-specified breakpoints to identify optimal change points in the nursing density series (24). We compared models with zero, one, and two breakpoints using Bayesian Information Criterion (BIC) and reported 95% confidence intervals for the estimated breakpoint positions (26). To assess whether any identified breakpoint was a denominator artifact from the 2009 population rebasing, we repeated the joinpoint analysis on absolute nursing personnel counts (without population denominator) and on physician density (which shares the same denominator), and compared the resulting breakpoints.

#### 2.5.2 Primary analysis

ITSA with GLS-AR(2) errors. Given the expected autocorrelation in annual workforce data, confirmed by the Durbin-Watson test on OLS residuals (DW = 0.96, *p* < 0.001), we specified a GLS model with second-order autoregressive errors as the primary analysis, estimated by maximum likelihood. The autoregressive order was selected by likelihood-ratio test against a first-order structure (L. Ratio = 10.84, *p* = 0.001) and was independently indicated by exhaustive Akaike Information Criterion (AIC) search in the forecasting model; residual diagnostics are reported in the Results. Estimates from the first-order specification are retained in Table 1 for comparison. The intervention estimates are invariant to the choice of time origin, and the baseline is therefore coded at 2003. We estimated the segmented regression model

**Table 1:** Interrupted time series analysis of nursing workforce density in Kazakhstan, 2003 to 2024.

Variable	GLS-AR(2) coefficient (primary)	SE	*p* value	GLS-AR(1) coefficient	SE	*p* value	OLS+NW coefficient	SE	*p* value
Intercept (baseline, 2003)	74.98	1.02	< 0.001	75.37	1.97	< 0.001	74.79	1.15	< 0.001
Time (pre-2014 trend/year)	+2.41	0.17	< 0.001	+2.50	0.30	< 0.001	+2.50	0.23	< 0.001
Intervention 2014 (level change)	−5.45	1.41	0.001	−8.20	2.16	0.001	−6.87	2.36	0.009
Time after 2014 (slope change)	−2.16	0.25	< 0.001	−2.18	0.49	< 0.001	−2.20	0.30	< 0.001

GLS-AR(2): GLS with second-order autoregressive errors, estimated by maximum likelihood; phi1 = 0.76, phi2 = −0.68; AIC = 90.7. The autoregressive order was selected by likelihood-ratio test against the first-order specification (L. Ratio = 10.84, p = 0.001) and was independently indicated by exhaustive AIC search in the forecasting model. GLS-AR(1): the first-order specification, retained for comparison; phi = 0.57; AIC = 99.5. OLS+NW: ordinary least squares with Newey-West heteroscedasticity and autocorrelation consistent standard errors (lag = 2, Andrews bandwidth); R-squared = 0.93. The post-intervention net trend equals the sum of the time and slope-change coefficients (GLS-AR(2): +2.41 + (−2.16) = +0.25 per 10,000 per year). The slope-change estimate was invariant to the error specification, whereas the level-change estimate attenuated by roughly one third under AR(2). Intervention indicator equals 1 for years from 2014 onward. SE, standard error. N = 22 annual observations.

$$\begin{array}{l} \text{Y}\left(\text{t}\right)=\text{B}0+\text{B}1^{*}time+\text{B}2^{*}intervention+\text{B}3^{*}time\text{\_}after+e\left(t\right), \end{array}$$

where time is a counter equal to 0 in 2003 and incrementing by one each year; intervention equals 1 from 2014 onward and 0 otherwise; time_after equals the number of years elapsed since 2014, multiplied by the intervention indicator; B0 is the baseline level, B1 the pre-intervention trend, B2 the immediate level change at 2014, B3 the slope change, and e(t) the error term with second-order autoregressive structure (20, 21).

We also report OLS estimates with Newey-West heteroscedasticity and autocorrelation consistent (HAC) standard errors (SE), using bandwidth selected by the Andrews (1991) formula, for comparability with the existing ITSA literature (27, 28).

Secondary and sensitivity analyses. We added a binary covariate for the 2007 bachelor introduction to control for potential confounding. We fitted an ARIMA model with intervention regressors for forecasting, selecting model order automatically by AIC (29). We performed six sensitivity analyses: (S1) absolute personnel counts; (S2) log-transformed density; (S3) nurse-to-physician ratio; (S4) excluding 2020 and 2021 to test whether the trend reversal was sensitive to the pandemic-period observations; (S5) an exploratory two-intervention model in which the 2007 reform enters with both a level and a slope component, alongside the 2014 intervention; and (S6) an alternative intervention point at 2017, when formal State Compulsory Educational Standards (GOSO) standards were approved and large-scale implementation began. The density-scale analyses (S4, S5, S6) used GLS-AR(2), as in the primary model. The transformed-scale analyses (S1, S2, S3) used OLS with Newey-West standard errors, a conservative robust estimator that makes no parametric assumption about the form of the serial dependence.

Regional analysis. We calculated nursing density for each of 20 administrative regions for 2003 to 2024 and assessed inequality using the coefficient of variation and the maximum-to-minimum density ratio. Administrative boundary changes in 2009, 2018, and 2022 affect the comparability of regional metrics over time and are noted where relevant. A hierarchical specification was not adopted because regional boundaries changed during the study period, and a random-effects structure would partly reflect administrative reorganization rather than workforce dynamics.

### 2.6 Software

All analyses were performed in R version 4.5.0 (R Foundation for Statistical Computing, Vienna, Austria) using the following packages: nlme (version 3.1-168) for generalized least squares estimation, lmtest (0.9–40) and sandwich (3.1–0) for robust standard errors, car (3.1–3) for collinearity diagnostics, forecast (9.0.2) for ARIMA modeling, and segmented (2.2–1) for joinpoint regression. The dataset and full analysis script are openly available in Zenodo (https://doi.org/10.5281/zenodo.20757110), together with documentation and session information.

## 3 Results

### 3.1 National trends

Between 2003 and 2024, the absolute number of nursing and midwifery personnel in Kazakhstan increased from 115,006 to 191,728, a gain of 67%. Over the same period, the population grew from 14.9 to 20.0 million (34%), resulting in a more modest increase in workforce density from 77.4 to 95.7 per 10,000 population (24%).

The density trajectory followed a distinct pattern (Figure 1A). From 2003 to 2012, density rose steadily from 77.4 to a peak of 101.2 per 10,000. After 2012, density declined and then stabilized, fluctuating between 93.3 and 100.0 through 2024.

**Figure 1:**
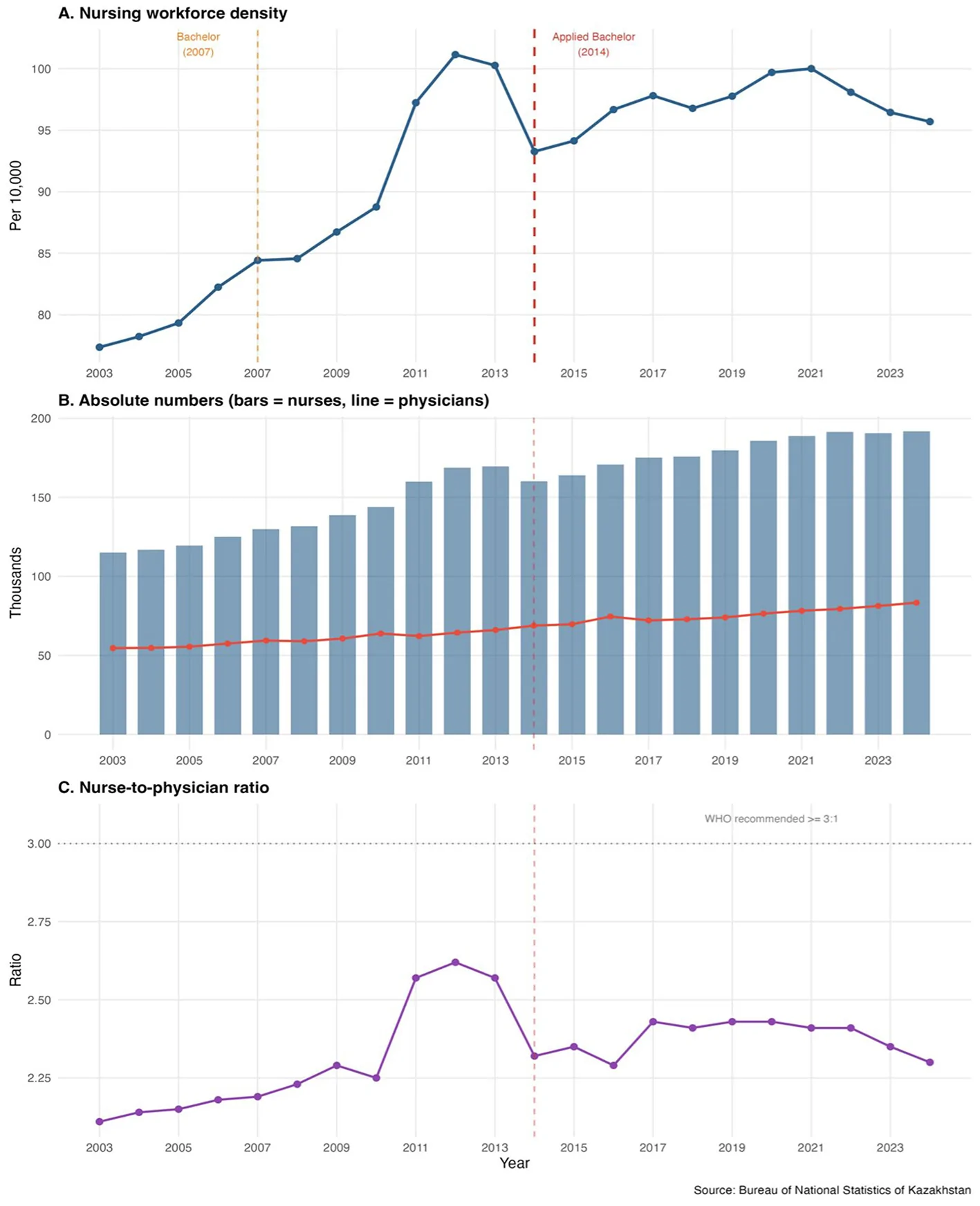
Nursing workforce indicators in Kazakhstan, 2003 to 2024. **(A)** Nursing and midwifery personnel density per 10,000 population. **(B)** Absolute numbers of nursing personnel (bars) and physicians (line), in thousands. **(C)** Nurse-to-physician ratio. Orange dashed line: introduction of bachelor-level nursing programmes (2007). Red dashed line: initiation of the Applied Bachelor of Nursing programme (2014). Horizontal dotted line in **(C):** WHO-recommended minimum nurse-to-physician ratio of 3:1.

Physician density increased more consistently from 36.7 to 41.6 per 10,000 (Figure 1B). The nurse-to-physician ratio peaked at 2.62 in 2012, then declined to 2.30 by 2024, remaining below the WHO-recommended threshold of 3:1 throughout the study period (Figure 1C).

Table 2 summarizes workforce indicators by reform period. Mean nursing density was 79.3 per 10,000 (SD 2.1) in the pre-reform period (2003 to 2006), 91.9 (SD 7.4) in the post-bachelor period (2007 to 2013), and 96.9 (SD 2.1) in the post-applied bachelor period (2014 to 2024). The notably higher SD in the 2007 to 2013 period reflects the rapid growth and subsequent deceleration within that phase, consistent with the joinpoint finding reported below.

**Table 2:** Nursing workforce indicators in Kazakhstan by reform period, 2003 to 2024.

Period	*N* (years)	Nursing density, mean (SD^*^)	Range	Physician density, mean	*N*:*P* ratio^**^, mean	Population, million
Pre-reform (2003–2006)	4	79.3 (2.1)	77.4–82.2	37.0	2.14	15.0
Post-bachelor (2007–2013)	7	91.9 (7.4)	84.4–101.2	38.5	2.39	16.2
Post-applied bachelor (2014–2024)	11	96.9 (2.1)	93.3–100.0	40.8	2.38	18.5

Nursing density and physician density are expressed per 10,000 population.

* SD-standard deviation. ^**^N:P ratio-nurse-to-physician ratio.

Pre-reform: before introduction of bachelor-level nursing education.

Post-bachelor: after introduction of university bachelor programmes.

Post-applied bachelor: after initiation of the Applied Bachelor of Nursing programme.

### 3.2 Joinpoint analysis

The data-driven segmented regression identified a single breakpoint in nursing density at approximately 2012 (95% CI: 2010 to 2014; BIC = 113.3), 2 years before the 2014 reform. Before 2012, nursing density increased by approximately 2.4 per 10,000 per year. After 2012, the trend reversed. The two-breakpoint model placed changes at 2010 and 2011 (BIC = 109.3). Although its BIC was marginally lower, the two estimated joinpoints fell in adjacent years, leaving a segment of approximately one observation, which is below the minimum segment length required for a separately identified slope. The one-breakpoint model was therefore retained as the primary specification; both models place the structural change within the same period, 2010 to 2012.

To assess whether this breakpoint was a denominator artifact from the 2009 population rebasing, we repeated the joinpoint analysis on absolute nursing personnel counts. The breakpoint in absolute numbers was also identified at approximately 2012 (95% CI: 2009 to 2015), confirming that the trend change was not driven by changes in the population denominator. In contrast, joinpoint regression applied to physician density identified a breakpoint at approximately 2016, a different year, indicating that the 2012 turning point was specific to the nursing workforce and not a shared denominator effect. The 95% confidence interval for the 2012 breakpoint (approximately 2010 to 2014) does not exclude 2014, so these data alone cannot statistically rule out that the structural change coincided with the reform. Three independent observations nonetheless make a 2014 causal effect unlikely. First, the point estimate of the breakpoint (2012) precedes the reform by 2 years. Second, the same 2012 breakpoint was recovered in absolute personnel counts (95% CI approximately 2009 to 2015), independent of the population denominator, whereas physician density showed a distinct breakpoint near 2016. Third, the first cohort of Applied Bachelor graduates did not complete training until 2018, 4 years after the reform was initiated, so an immediate 2014 supply-side effect is implausible on training-cycle grounds.

### 3.3 Interrupted time series analysis

#### 3.3.1 Primary analysis [GLS-AR(2)]

The GLS model with second-order autoregressive errors estimated a pre-2014 trend of +2.41 per 10,000 per year, a level change of minus 5.45 at 2014 (*p* = 0.001), and a slope change of minus 2.16 per year (*p* < 0.001), yielding a net post-2014 trend of +0.25 per year (Table 1). The autoregressive parameters (phi1 = 0.76, phi2 = minus 0.68) describe a stationary second-order error process; the moduli of the characteristic roots (1.212) confirm stationarity. Normalized residuals showed no remaining autocorrelation (Ljung-Box *p* = 0.97, 0.99, 0.38 and 0.61 at lags 1, 2, 3 and 5) and were consistent with normality (Shapiro-Wilk W = 0.970, *p* = 0.709); autocorrelation and partial autocorrelation plots are provided as Supplementary Figure S1. The GLS-AR(2) model achieved the lowest AIC (90.7) among the segmented-regression specifications (Table 3). The slope change was essentially invariant to the error specification [minus 2.18 under AR(1), minus 2.16 under AR(2)], whereas the level coefficient attenuated by roughly one third, reinforcing the interpretation that the level coefficient is model-dependent and should not be read as a substantive effect.

**Table 3:** Model comparison for interrupted time series analysis.

Model	AIC	BIC	Key feature
Null (linear trend only)	132.5	135.7	No intervention
OLS + Newey-West	103.8	109.2	HAC standard errors
With 2007 covariate (OLS)	102.7	109.3	Controls for bachelor reform
GLS-AR(1)	99.5	106.1	First-order autoregressive errors
GLS-AR(2; primary)	90.7	98.3	Second-order autoregressive errors; lowest AIC and BIC
S6: 2017 intervention (OLS)	120.0	125.4	Alternative intervention point

AIC, Akaike Information Criterion; BIC, Bayesian Information Criterion; lower values indicate better fit. GLS-AR(2) is the primary model. All models with the 2014 intervention substantially improved fit over the null. The ARIMA(2, 0, 0) forecasting model is reported in the forecasting section; its likelihood is not directly comparable with the segmented-regression fits and it is therefore not included in this comparison.

#### 3.3.2 OLS comparison

The OLS model with Newey-West standard errors produced consistent estimates (level: minus 6.87, SE 2.36, *p* = 0.009; slope: minus 2.20, SE 0.30, *p* < 0.001; R-squared = 0.93). Residuals passed the Shapiro-Wilk normality test (W = 0.96, *p* = 0.51). Figure 2 shows the observed values, fitted segments, and counterfactual projection.

**Figure 2:**
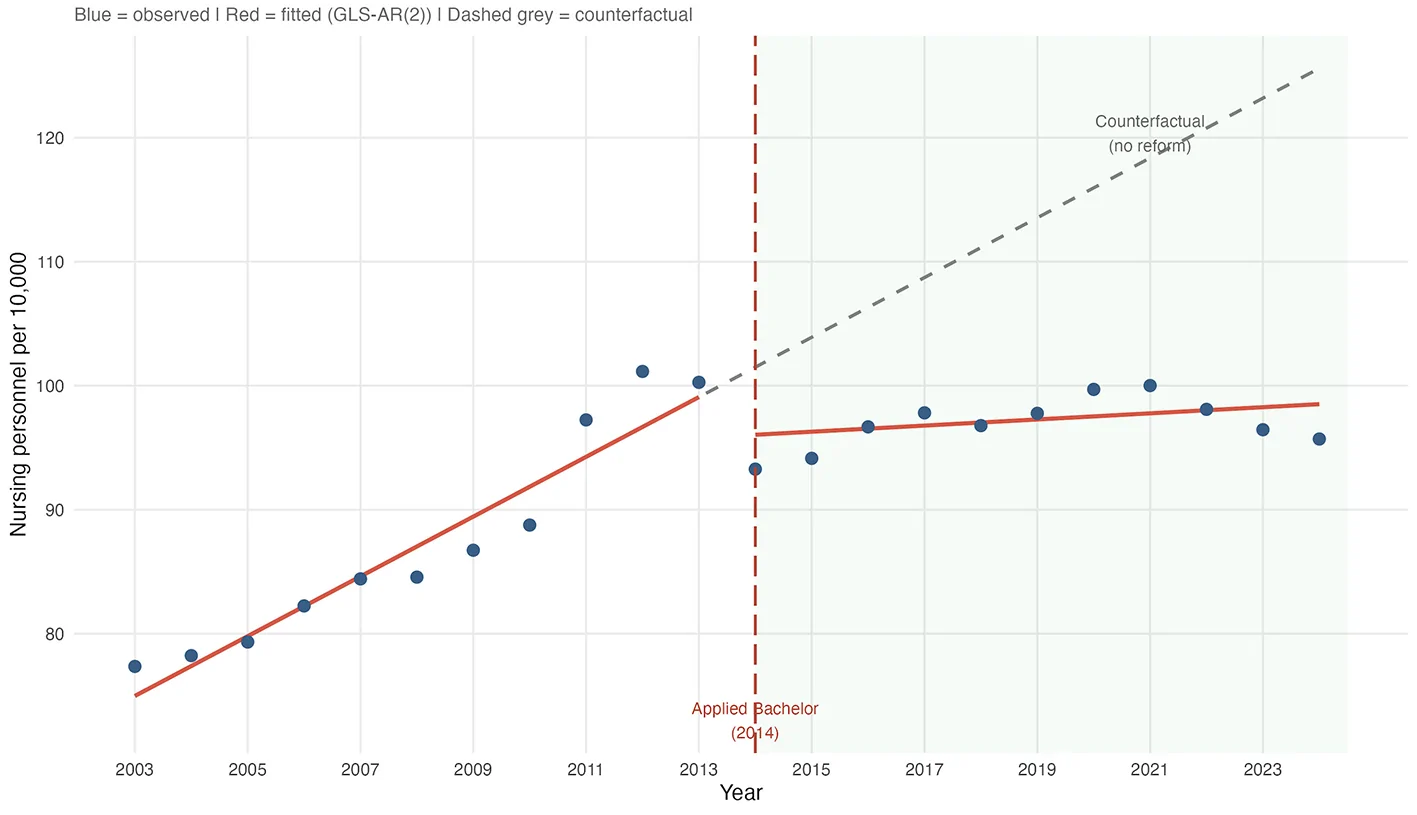
Interrupted time series analysis of nursing workforce density in Kazakhstan, 2003 to 2024. Blue points: observed values. Red line: fitted segmented regression [GLS-AR(2) primary specification]. Gray dashed line: counterfactual trajectory based on the pre-2014 trend. Green shading: post-intervention period. Vertical dashed line: 2014 intervention point. Source: Bureau of National Statistics of Kazakhstan.

#### 3.3.3 Reconciling ITSA and joinpoint results

The ITSA model produced statistically significant coefficients at the 2014 intervention point. However, the joinpoint analysis placed the structural breakpoint at 2012 (95% CI: 2010 to 2014), 2 years earlier. The significant ITSA coefficients therefore reflect the proximity of the pre-specified hinge point to the actual data-driven trend reversal rather than a causal effect of the reform. In segmented regression, placing the hinge near a genuine breakpoint will produce significant coefficients regardless of whether any policy at that date caused the change. The level change coefficient (B2) should be interpreted as a statistical parameter of the model, not as evidence of an immediate workforce reduction following the ministerial order. This interpretation is further supported by the S6 sensitivity analysis (2017 intervention), in which the slope change remained significant (minus 2.14, *p* = 0.001) but the level shift was not (minus 2.43, *p* = 0.27), consistent with a gradual rather than abrupt transition. An additional consideration is the lag between policy initiation and workforce effect: the first cohort of Applied Bachelor graduates completed their training in 2018, meaning that any supply-side effect of the 2014 reform could not have appeared in workforce data before 2018 to 2019 at the earliest. This lag further supports using the joinpoint result as the primary evidence on when the structural trend change actually occurred. When the hinge was placed at the data-driven 2012 breakpoint, no significant level shift was detected (minus 0.34, *p* = 0.86) while the slope change remained significant (minus 2.27, *p* < 0.001), consistent with a gradual trend reversal rather than an abrupt change and indicating that the significant level coefficient at 2014 reflects hinge placement. A hinge set at 2017, as in S6, falls within the post-deceleration segment, so a segmented model will detect a difference in slope relative to the pooled earlier period even when the underlying change point lies before it; this is a further illustration of the sensitivity of segmented parameters to hinge placement rather than evidence of a distinct 2017 effect.

#### 3.3.4 Model robustness

In the secondary analysis, in which the 2007 reform enters as a level shift only, the 2014 estimates were a level change of minus 8.44 (*p* < 0.001) and a slope change of minus 2.70 (*p* < 0.001); the 2007 covariate showed a marginally significant negative level shift (minus 3.93, *p* = 0.088). This coefficient has no mechanistic interpretation in terms of workforce supply, since no cohort could enter or leave the labor market in the year the programme was introduced, and it is best read as an adjustment for the concurrent policy environment. The two-intervention model reported as S5 in Table 4, in which the 2007 reform enters with both level and slope components, yields different 2014 estimates (level minus 7.07, slope minus 2.67) and is presented as exploratory: variance inflation factors (VIF) for the temporal terms are high (177.1 for the baseline time variable and 169.9 for the 2007 slope term), which is intrinsic to segmented parameterizations containing multiple time-varying components rather than a defect of estimation. The correlation between the 2014 level coefficient and the remaining coefficients does not exceed 0.38 in absolute value, indicating that the 2014 estimates remain identifiable. All models with the 2014 intervention substantially improved fit over the null linear trend (AIC 132.5; Table 3). Excluding the 2020 and 2021 observations produced a level change of minus 5.48, nearly identical to the primary estimate. In that analysis the correlation structure was conditioned on the time covariate, so that the 2-year gap is encoded in the correlation matrix rather than treated as contiguous; refitting with a continuous-time correlation structure returned identical estimates.

**Table 4:** Sensitivity analyses.

Analysis	Level coefficient	*p* value	Slope coefficient	*p* value	Outcome
Primary GLS-AR(2) model	−5.45	0.001	−2.16	< 0.001	Density per 10,000
S1: absolute numbers	−10,431	0.031	−2,569	< 0.001	Personnel count
S2: log-transformed	−0.077	0.004	−0.025	< 0.001	Log(density)
S3: nurse: physician ratio	−0.247	0.003	−0.050	< 0.001	*N*:*P* ratio
S4: excluding 2020–2021	−5.48	0.002	−2.24	< 0.001	Density per 10,000
S5: two-intervention model (2007 level and slope; 2014), exploratory	−7.07	< 0.001	−2.67	< 0.001	Density per 10,000
S6: intervention at 2017	−2.43	0.267	−2.14	0.001	Density per 10,000

Primary, S4, S5, and S6 were estimated by GLS with second-order autoregressive errors (GLS-AR(2), maximum likelihood), sharing the original density scale (phi1/phi2: primary 0.76/−0.68; S4 0.68/−0.63; S5 0.63/−0.71; S6 0.93/−0.57). S1-S3 were estimated by ordinary least squares with Newey-West standard errors (Andrews bandwidth, lag = 2), a conservative robust alternative for outcomes on transformed scales. Level coefficient: immediate change at the intervention point. Slope coefficient: change in annual trend after the intervention. S6 showed no significant level shift (p = 0.27) but a significant slope change (p = 0.001), consistent with a gradual rather than abrupt structural transition.

### 3.4 Forecasting

The ARIMA(2, 0, 0) model with intervention regressors projected nursing density to reach 111.0 per 10,000 by 2035 (95% prediction interval: 92.6 to 129.4; Table 5; Figure 3). The wide prediction interval reflects genuine uncertainty about future trends; the projection assumes continuation of current policy conditions. Full annual projections are provided in Supplementary Table S3.

**Table 5:** ARIMA forecast of nursing workforce density to 2035 (selected years).

Year	Point forecast	80% prediction interval	95% prediction interval
2025	95.9	92.9 to 98.8	91.3 to 100.4
2027	97.9	90.4 to 105.3	86.4 to 109.3
2030	102.7	91.8 to 113.6	86.0 to 119.3
2033	107.8	95.9 to 119.6	89.6 to 125.9
2035	111.0	98.9 to 123.0	92.6 to 129.4

ARIMA(2, 0, 0) with intervention and time-after-intervention as external regressors. Values are nursing and midwifery personnel per 10,000 population. Full annual projections are in Supplementary Table S3.

**Figure 3:**
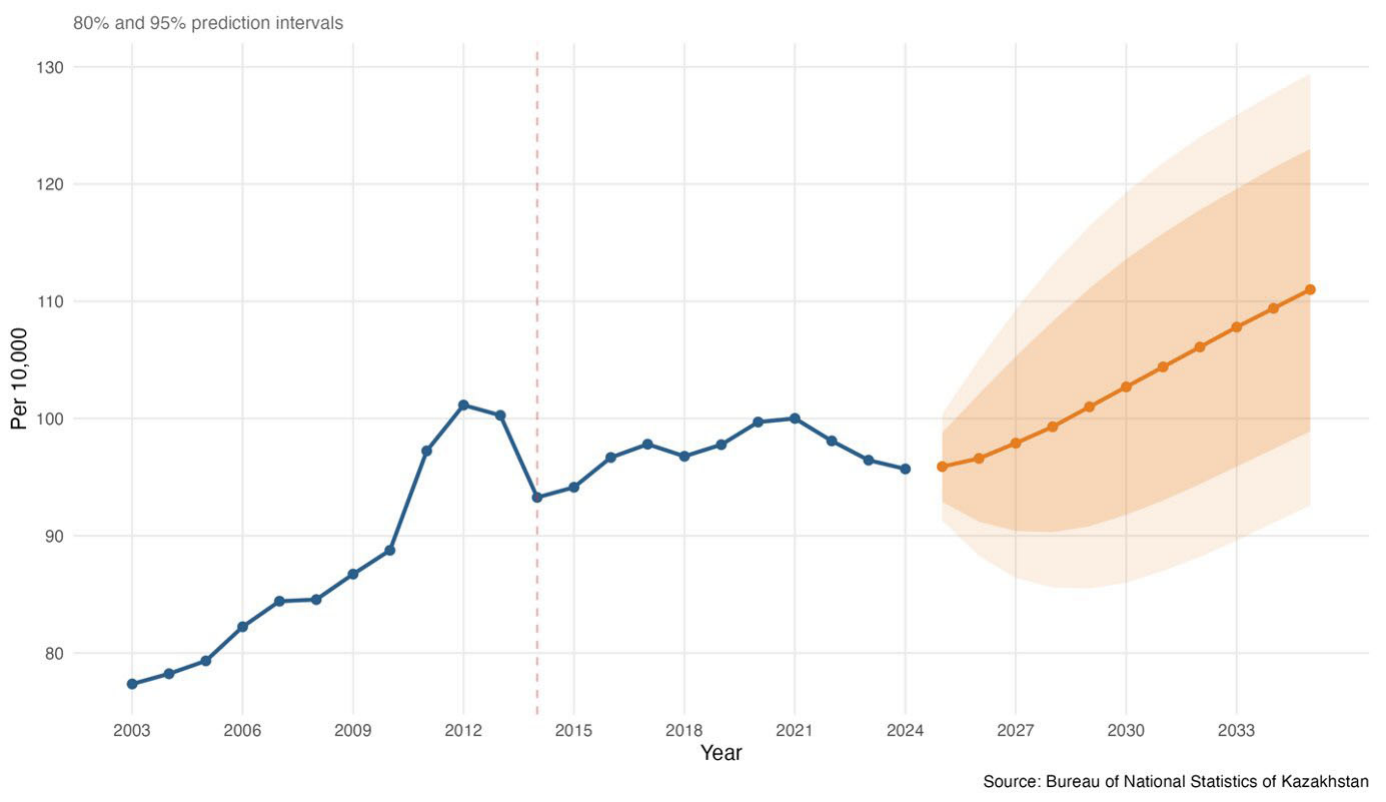
ARIMA(2, 0, 0) forecast of nursing workforce density to 2035. Blue line and points: observed values (2003 to 2024). Orange line and points: point forecast (2025 to 2035). Dark orange shading: 80% prediction interval. Light orange shading: 95% prediction interval. Vertical dashed line: 2014 intervention point. Source: Bureau of National Statistics of Kazakhstan.

### 3.5 Regional analysis

All 20 current administrative regions had complete data for 2024, accounting for 100% of the national nursing workforce. Nursing density in 2024 ranged from 72.0 per 10,000 in Almaty region to 134.8 in Kyzylorda, a 1.9-fold difference (Table 6). Note that Almaty region and Almaty city are distinct administrative units; the region excludes the city and has consistently lower nursing density.

**Table 6:** Nursing workforce density by administrative region of Kazakhstan, 2024.

Region	Nursing personnel	Physicians	Population	Nursing density	Physician density	*N*:*P* ratio
Kyzylorda	11,346	3,070	841,931	134.8	36.5	3.70
Abay	6,644	2,747	607,589	109.4	45.2	2.42
Ulytau	2,399	703	221,584	108.3	31.7	3.41
East Kazakhstan	7,713	2,906	727,053	106.1	40.0	2.65
West Kazakhstan	7,142	2,301	693,262	103.0	33.2	3.10
Aktobe	9,597	4,522	939,405	102.2	48.1	2.12
Zhambyl	12,445	3,655	1,222,593	101.8	29.9	3.40
Turkestan	21,650	6,806	2,142,171	101.1	31.8	3.18
Mangystau	7,904	2,609	786,841	100.5	33.2	3.03
North Kazakhstan	5,230	1,705	530,089	98.7	32.2	3.07
Karaganda	11,133	5,801	1,135,351	98.1	51.1	1.92
Astana city	13,688	10,540	1,430,105	95.7	73.7	1.30
Zhetysu	6,604	1,813	697,987	94.6	26.0	3.64
Shymkent city	11,261	5,614	1,222,066	92.2	45.9	2.01
Pavlodar	6,913	3,184	753,934	91.7	42.2	2.17
Atyrau	6,303	2,211	704,074	89.5	31.4	2.85
Almaty city	19,920	14,681	2,228,675	89.4	65.9	1.36
Akmola	6,244	2,082	787,981	79.2	26.4	3.00
Kostanay	6,567	2,536	829,984	79.1	30.6	2.59
Almaty region	11,025	3,893	1,531,167	72.0	25.4	2.83

Nursing density and physician density are expressed per 10,000 population. N:P ratio, nurse-to-physician ratio. Regions are ordered by nursing density from highest to lowest. Abay, Zhetysu, and Ulytau regions were created in 2022. Turkestan region and Shymkent city were created in 2018 (data for earlier years correspond to the former South Kazakhstan region). Source: Bureau of National Statistics of Kazakhstan.

Regions fell into three broad groups (Figure 4). Kyzylorda and East Kazakhstan consistently had the highest densities. Almaty region, Kostanay, and Akmola had the lowest. The three cities of republican significance (Astana, Almaty, Shymkent) occupied middle positions for nursing density but had the lowest nurse-to-physician ratios (1.30 in Astana, 1.36 in Almaty city), reflecting their high concentration of physicians.

**Figure 4:**
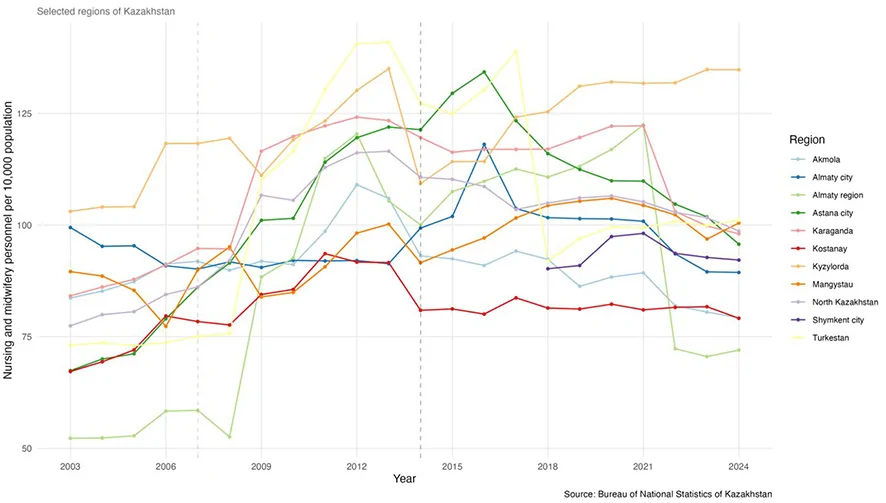
Regional variation in nursing and midwifery workforce density in selected regions of Kazakhstan, 2003 to 2024. Values are nursing and midwifery personnel per 10,000 population. Gray dashed line: 2014 intervention point. Orange dashed line: 2007 bachelor programme introduction.

The coefficient of variation across regions was 15 to 16% from 2003 to 2008, increased to 21 to 27% from 2009 to 2021, and then dropped to 13 to 14% from 2022 to 2024. The 2009 increase coincided with the population rebasing, and the 2022 decrease reflected the creation of three new administrative regions. Regional inequality metrics should therefore be interpreted with caution across the full study period.

The concurrent consolidation of the hospital network provides structural context. The number of hospital organizations in Kazakhstan declined from 1,029 in 2003 to a minimum of 749 in 2019 (minus 27%), before recovering to 830 in 2024. After 2014, hospital numbers decreased by a further 162 (minus 17.8%) through 2019, compressing the absorptive capacity of the health system for new graduates. Regions with the steepest hospital closures, including East Kazakhstan (minus 40% on harmonized 2022 boundaries, i.e., combining the present East Kazakhstan and Abai regions; the unadjusted figure of minus 72% reflects the 2022 split of Abai) and Turkestan (minus 28% on harmonized 2018 boundaries, combining Turkestan region and Shymkent city; the unadjusted minus 59% reflects the 2018 split of Shymkent), experienced simultaneous consolidation of nursing staff into fewer, larger facilities (Supplementary Table S2).

## 4 Discussion

This study examined 22 years of nursing workforce data in Kazakhstan and produced three principal findings. First, nursing density in Kazakhstan grew from 77.4 to a peak of 101.2 per 10,000 between 2003 and 2012, then stagnated near 95 to 100 through 2024. Second, joinpoint regression placed the structural breakpoint at approximately 2012 (95% CI: 2010 to 2014), 2 years before the 2014 educational reform. Sensitivity analyses confirmed that this breakpoint was not a denominator artifact: the same year was identified in absolute nursing counts, while physician density showed a distinct breakpoint at 2016. Third, although the ITSA model produced statistically significant coefficients at 2014, these reflected the proximity of the pre-specified hinge to the actual data-driven trend reversal rather than a causal reform effect. The deceleration of nursing workforce growth therefore preceded the reform and is consistent with structural factors operating before policy implementation.

### 4.1 Interpretation of the 2012 breakpoint

Several mechanisms could explain the 2012 turning point. None can be directly quantified with the available data, and we present them in descending order of plausibility rather than as parallel, equally weighted explanations.

The most parsimonious interpretation is that the 2003 to 2012 growth phase represented recovery from the workforce contraction of the 1990s, when the absolute number of nurses fell by approximately half (4, 5). This pattern is observable across the post-Soviet region: the workforce reconstitution of the 2000s was followed by demographic plateaus once trained personnel returned to the system (8, 18).

The 2007 introduction of bachelor-level nursing programmes lengthened the training cycle, and pipeline effects of such reforms usually take several years to manifest in workforce data, corresponding to the 2012 turning point. Comparable pipeline lags have been documented internationally. The United Kingdom's Project 2000 transition from hospital-based diploma to higher-education preparation was followed by a substantial reduction in student nurse numbers and persistent recruitment difficulties in the years after implementation (30). Across the European Higher Education Area, the Bologna Process produced uneven nursing workforce trajectories, with Germany, Poland, the Czech Republic and the United Kingdom experiencing sustained shortages after the educational transition (31–33).

Two further mechanisms are more plausibly reinforcing than primary. The number of hospital organizations in Kazakhstan declined from 1,029 in 2003 to 749 in 2019, a 27% reduction concentrated mainly in 2014 to 2019, when the annual rate of decline was roughly three times that of the 2003 to 2014 period. Consolidation reduced the number of available nursing positions, particularly in rural areas, and altered the institutional capacity to absorb new graduates (15, 18). This demand-side contraction reinforced any supply-side pipeline effect. Alongside this, outflow of nurses to higher-wage labor markets, particularly the Russian Federation, has been documented across Central Asia and the Caucasus (18). We could not directly quantify migration in this study, but it remains a plausible contributing mechanism that would manifest as a deceleration in observed density even in the absence of changes in domestic graduate output.

Macroeconomic conditions require closer examination, since economic downturns typically reach health workforce data with a lag, through hiring freezes, salary restraint and reduced absorption of graduates (34, 35), and the timing of the 2012 turning point is in principle compatible with a delayed response to the 2008 global financial crisis. The Kazakhstani experience, however, diverged from the European pattern. Real GDP growth slowed in 2008 and was marginally negative in 2009, but the government ran a budget surplus in every year from 2008 to 2011, public spending on health more than doubled between 2007 and 2011, and 61 hospitals were built between 2008 and 2012 under a national construction programme (36). Health sector employment was therefore shielded from the austerity mechanisms documented elsewhere, and on the available evidence the 2008 crisis is unlikely to have been a primary driver of the 2012 deceleration. The later devaluations of the tenge in February 2014 and again in 2015, in response to falling oil prices and regional currency pressures, reduced the purchasing power of tenge-denominated health sector wages relative to dollar-equivalent earnings abroad. The rouble, however, depreciated more sharply than the tenge during 2014 and 2015, temporarily reducing rather than increasing the relative wage incentive for migration to the Russian Federation; this channel is more plausibly relevant in the subsequent period of currency stabilization (37). We cannot directly attribute density changes to these macroeconomic shocks, and their timing follows rather than precedes the breakpoint.

Finally, we considered the possibility that the 2012 breakpoint reflected administrative or definitional changes in how the Bureau of National Statistics records mid-level medical personnel. We found no documented changes in indicator code 631502 around 2012, and the breakpoint persisted in absolute numbers (independent of population denominator) and was distinct from the breakpoint identified for physicians (which used the same indicator and denominator structure). These checks support a real workforce trend rather than a reporting artifact.

Taken together, recovery exhaustion and the pipeline effect of the 2007 reform are the most parsimonious, as both predict a turning point in the early 2010s without an external shock. Hospital consolidation and migration likely acted as reinforcing rather than primary drivers, since their documented timing follows the breakpoint. The macroeconomic and statistical-reporting explanations are least supported, the former because the 2008 crisis did not translate into health sector austerity in Kazakhstan and the 2014 to 2015 devaluations follow the breakpoint, and the latter because it was directly tested and rejected. The available data cannot formally separate these mechanisms, and this ranking is an interpretive judgment rather than a statistical result.

The transition to bachelor-level nursing education has progressed slowly. Official Ministry of Health data indicate that more than 20,000 nurses with applied bachelor diplomas have been employed in healthcare facilities over the 6 years preceding 2025, representing approximately 14% of the current nursing workforce of 146,000 (25). A cross-sectional survey of 2,263 nurses across Kazakhstan found that 75.3% held nurse practitioner roles, but the proportion with bachelor-level qualifications remained limited (38). This contrasts with the situation in high-income countries, where bachelor-level preparation is standard for newly registered nurses (2). Even among nurses who hold Applied Bachelor qualifications, adoption of the expanded scope of practice has been incomplete: a survey of 280 primary care nurses in Astana and Ust-Kamenogorsk found that only 39% and 53%, respectively, performed independent patient consultations within their competence, and fewer than half performed health promotion functions (39). This gap between educational qualification and clinical practice helps explain why the formal expansion of bachelor-level training has not yet translated into measurable workforce density gains.

### 4.2 International and regional comparisons

Kazakhstan's nursing density of 95.7 per 10,000 in 2024 exceeds the WHO European Region average of approximately 80 per 10,000 (40) but falls below high-income European countries such as Finland (145), Norway (174), and Switzerland (178) (41). These comparisons should be interpreted with caution. International density figures generally reflect registered nurses (ISCO 2221), whereas the Kazakhstan indicator is composite, including nurses, midwives and feldshers. Available official data indicate that nurses constitute approximately 77% of this composite category as of 2025 (25), Comparable figures are not published for earlier years, so the stability of this proportion across the study period cannot be verified. No administrative reorganization of the constituent cadres is documented for the period, and the joinpoint analysis of absolute personnel counts identifies the same structural change as the density analysis, which argues against a compositional artifact; these observations provide partial reassurance rather than evidence of stability. Absolute international comparisons should therefore be treated as indicative.

Within Central Asia, Kazakhstan has the highest nursing density (18). The trajectory we describe also resembles patterns documented in Eastern European countries that transitioned from vocational to bachelor-level nursing education during the Bologna Process. Studies from Poland, the Czech Republic, and Lithuania have reported persistent post-reform workforce shortages alongside growing migration to Western European countries (31, 32). The United Kingdom experienced an analogous transitional shortage after Project 2000 raised pre-registration training to higher-education status; recruitment difficulties persisted for over a decade (30). A parallel analysis of the Kazakhstan ophthalmology workforce found that the introduction of 3-year residency training in 2008 was associated with a temporary reduction in rural ophthalmologist density, interpreted as a pipeline effect of longer training programmes (22). These convergent findings across professions and countries suggest that the workforce trajectory observed in Kazakhstan is not unique but consistent with a broader pattern of transition-period stagnation following educational upgrading.

The nurse-to-physician ratio of 2.30 in 2024 places Kazakhstan below most OECD countries and below the WHO 3:1 threshold. Countries with strong primary care systems maintain ratios of 3.0 to 4.7 (41). The declining ratio reflects faster physician workforce growth, a pattern also observed in other post-Soviet states (6, 18). The 1.9-fold regional variation in density (72.0 to 134.8) represents a persistent structural challenge. Almazan et al. (42) found that job satisfaction among Kazakh nurses varied substantially by practice environment, suggesting that retention factors operate alongside recruitment in shaping regional disparities.

### 4.3 Implications for nursing workforce policy

First, the stagnation of density despite increasing absolute numbers signals that current educational output cannot keep pace with population growth of approximately 400,000 per year. Our ARIMA forecast (111.0 per 10,000 by 2035; 95% prediction interval: CI: 92.6 to 129.4) depends on sustaining current output and retention rates. Expanding training capacity, particularly in the Applied Bachelor track, will be essential. Comparable expansion has been pursued in countries that experienced post-Bologna shortages, often through accelerated apprenticeship and conversion programmes that bring practicing vocational nurses into degree-level qualifications (32). Currently, 94 medical colleges (22 state and 72 private), including 39 designated as higher medical colleges, train more than 23,000 mid-level specialists annually (25). Aligning educational output with population growth will require expanding capacity in the applied bachelor track and improving graduate retention in the workforce.

Second, the transition to bachelor-level education remains a justified long-term investment despite the transitional pipeline costs. Aiken et al. (2) found that each 10% increase in the proportion of bachelor-prepared nurses was associated with 7% lower odds of surgical mortality across nine European countries. Kazakhstan's currently low proportion represents both a quality gap and an opportunity. The transition must be managed carefully to avoid prolonged supply shortfalls, as the Project 2000 experience in the United Kingdom demonstrated (30). Accelerated tracks for working nurses, as introduced in 2016, are an important mitigation measure (39).

Third, regional disparities require targeted action. Almaty region, Kostanay, and Akmola consistently had the lowest densities throughout the study period. Financial incentive schemes alone have produced only modest effects in Kazakhstan (14) and elsewhere in the post-Soviet region. Expanding educational capacity in underserved regions, strengthening clinical training infrastructure, and addressing workplace conditions are likely to be more effective when combined with financial measures (18, 32, 42).

Fourth, the declining nurse-to-physician ratio deserves explicit planning targets. As Kazakhstan introduces expanded practice roles for bachelor-prepared nurses (39), maintaining an adequate ratio becomes essential for task-sharing models to function. Workforce planning should set explicit targets for the nurse-to-physician ratio alongside density targets and educational composition targets. The risks of pursuing numeric workforce expansion without transparent forecasting and stakeholder participation have recently been documented in the region, where rapid quota-driven reform was found unlikely to resolve distributional gaps on its own (43).

### 4.4 Strengths and limitations

This study has several methodological strengths. We analyzed 22 years of nationally representative data covering the entire nursing and midwifery workforce. The balanced ITSA design meets minimum power recommendations (23). We combined hypothesis-driven ITSA with data-driven joinpoint regression and confirmed robustness through six sensitivity analyses and an alternative model specification. The joinpoint sensitivity analyses on absolute numbers and physician density addressed the population rebasing concern directly and supported the validity of the 2012 breakpoint.

The study has several limitations. First, this is an ecological study and we cannot draw causal inferences. The significant ITSA coefficients at 2014 do not establish that the reform caused the deceleration, as the joinpoint analysis demonstrated. Second, the indicator (code 631502) is a composite measure that includes nurses, midwives and feldshers. According to official Ministry of Health data, nurses constitute approximately 77% of this composite category, with 146,000 of the more than 190,000 mid-level medical personnel registered as nurses in 2025 (25). This proportion is consistent with cross-sectional survey data from Kazakhstan, where 75.3% of surveyed mid-level personnel were nurses in clinical roles (38). Comparable figures are not published for earlier years, so the stability of this proportion across the study period cannot be verified; the joinpoint analysis of absolute personnel counts identifies the same structural change, which argues against a compositional artifact. Absolute international comparisons with countries that report registered nurses only (ISCO 2221) should be interpreted with this categorical difference in mind. Third, administrative boundary changes in 2009, 2018, and 2022 affect the comparability of regional metrics across the full study period. Fourth, we lacked data on educational graduates, programme enrolment, attrition, and migration, which would have allowed direct testing of the pipeline mechanism. Fifth, the ARIMA projection extends 11 years beyond a 22-year observation series and is therefore contingent on the continuity of current policy conditions and on the absence of unmodelled macroeconomic or institutional shocks; it should be read as indicative of the trajectory implied by past trends rather than as a prediction. Sixth, because joinpoint confidence intervals were estimated from score-based statistics in a small sample, they should be interpreted as approximate.

## 5 Conclusions

Nursing workforce density in Kazakhstan grew rapidly from 2003 to 2012, then stagnated through 2024. Joinpoint regression placed the structural breakpoint at approximately 2012, 2 years before the 2014 Applied Bachelor reform, indicating that the deceleration preceded the reform and reflected the exhaustion of post-independence recovery rather than a causal reform effect. This pattern carries a methodological lesson beyond Kazakhstan: when a reform coincides with the tail of a prior recovery phase, a pre-specified ITSA point can absorb an earlier structural change and misattribute it to the reform; pairing ITSA with data-driven joinpoint regression guards against this. Although Kazakhstan's nursing density exceeds the WHO European Region average, the nurse-to-physician ratio has been declining and educational output is insufficient to keep pace with population growth. Sustained expansion of nursing programme capacity, accelerated conversion tracks for vocational nurses, targeted regional strategies, and explicit planning targets for the nurse-to-physician ratio are needed.

## Data Availability

The datasets presented in this study can be found in online repositories. The names of the repository/repositories and accession number(s) can be found below: https://doi.org/10.5281/zenodo.20757110.

## References

[1] World Health Organization. State of the World's Nursing 2020: Investing in Education, Jobs and Leadership. 1st ed. Geneva: World Health Organization. (2020).

[2] Aiken LH Sloane DM Bruyneel L Van den Heede K Griffiths P Busse R . Nurse staffing and education and hospital mortality in nine European countries: a retrospective observational study. Lancet. (2014) 383:1824–30. doi: 10.1016/S0140-6736(13)62631-8 24581683 PMC4035380

[3] Tamata AT Mohammadnezhad M A. systematic review study on the factors affecting shortage of nursing workforce in the hospitals. Nurs Open. (2023) 10:1247–57. doi: 10.1002/nop2.1434 36303066 PMC9912424

[4] World Bank Open Data. World Bank Open Data. Available online at: https://data.worldbank.org (Accessed April 30, 2026).

[5] Kulzhanov M Rechel B. Health Systems in Transition. WHO Regional Office for Europe on behalf of the European Observatory on Health Systems and Policies. (2007) 9:158.

[6] Gulis G Aringazina A Sangilbayeva Z Kalel Z Leeuw E de Allegrante JP. Population health status of the republic of Kazakhstan: trends and implications for public health policy. Int J Environmental Res Public Health (2021) 18:12235. doi: 10.3390/ijerph182212235 34831990 PMC8621160

[7] Semenova Y Lim L Salpynov Z Gaipov A Jakovljevic M. Historical evolution of healthcare systems of post-soviet Russia, Belarus, Kazakhstan, Kyrgyzstan, Tajikistan, Turkmenistan, Uzbekistan, Armenia, and Azerbaijan: a scoping review. Heliyon. (2024) 10:e29550. doi: 10.1016/j.heliyon.2024.e29550 38655295 PMC11036062

[8] Rechel B Ahmedov M Akkazieva B Katsaga A Khodjamurodov G McKee M. Lessons from two decades of health reform in Central Asia. Health Policy Plan. (2012) 27:281–7. doi: 10.1093/heapol/czr040 21609971

[9] OECD. Health at a Glance 2023: OECD Indicators. Health at a Glance (2023) 2023. doi: 10.1787/7a7afb35-en

[10] European Health Observatory. Health systems in action: Kazakhstan |European Observatory on Health Systems and Policies. Copenhagen: WHO Regional Office for Europe. (2024). Available online at: https://eurohealthobservatory.who.int/publications/i/health-systems-in-action-kazakhstan-2024 (Accessed April 30, 2026).

[11] Ministry of Health of the Republic of Kazakhstan. Comprehensive Plan for the Development of Nursing in the Republic of Kazakhstan until 2020. [Government document / normative legal act]. Astana: Ministry of Health of the Republic of Kazakhstan. (2014). Available online at: https://prg.kz/document/?doc_id=39860292 (Accessed June 17, 2026).

[12] Ministry of Health of the Republic of Kazakhstan. On amendments to Order No. 647 of 31 July 2015 approving state compulsory educational standards and curricula for medical and pharmaceutical specialties. (2017). Available online at: https://adilet.zan.kz/rus/docs/V1700015362 (Accessed June 17, 2026).

[13] Ministry of Health of the Republic of Kazakhstan. On Approval of the Rules for Nursing Care. (2020). Available online at: https://prg.kz/document/?doc_id=38088059 (Accessed June 17, 2026).

[14] Jobalayeva B Khismetova Z Glushkova N Kozhekenova Z Abzaliyeva A Berikuly D . The impact of incentive scheme on rural healthcare workforce availability: a case study of Kazakhstan. Hum Resour Health. (2024) 22:23. doi: 10.1186/s12960-024-00905-0 38605387 PMC11010400

[15] Jobalayeva B Glushkova N Khismetova Z Tanatarova G Zhagiparova Z Semenova Y. Past, current status, and future trends of the rural healthcare network in the Republic of Kazakhstan. Sci Rep. (2025) 15:26913. doi: 10.1038/s41598-025-11432-w 40707627 PMC12290056

[16] Akpanova T Rakhypbekov T Semenova Y Mussakhanova A Turgambayeva A Zhanaliyeva M . The impact of healthcare reforms on the epidemiology workforce in Kazakhstan: an interrupted time series analysis with predictive modeling of nationwide data sources from 1998 to 2022. Healthcare (2025) 13:170. doi: 10.3390/healthcare13020170 39857196 PMC11765252

[17] Shaltynov A Abenova M Baibussinova A Semenova Y Omarov N Tanatarova G . Inequality in the distribution and utilization of healthcare resources in Kazakhstan (2002–2023): a spatiotemporal analysis. Int J Env Res Public Health (2025) 22:1762. doi: 10.3390/ijerph22111762 41302708 PMC12652948

[18] Llop-Gironés A Azhymambetova GK Asanova AK Salomuddin Y Boynazarova MH Raupov FO . Building health systems resilience in Central Asia through nursing and midwifery: evidence to inform policy action. Hum Resour Health. (2024) 22:82. doi: 10.1186/s12960-024-00964-3 39696577 PMC11654295

[19] Bernal JL Cummins S Gasparrini A. Interrupted time series regression for the evaluation of public health interventions: a tutorial. Int J Epidemiol. (2017) 46:348–55. doi: 10.1093/ije/dyw098 PMC5407170 27283160

[20] Wagner AK Soumerai SB Zhang F Ross-Degnan D. Segmented regression analysis of interrupted time series studies in medication use research. J Clin Pharm Ther. (2002) 27:299–309. doi: 10.1046/j.1365-2710.2002.00430.x 12174032

[21] Penfold RB Zhang F. Use of interrupted time series analysis in evaluating health care quality improvements. Acad Pediatr. (2013) 13:S38–44. doi: 10.1016/j.acap.2013.08.002 24268083

[22] Semenova Y Urazhanova M Aimyshev T Gaipov A. Associations between medical education reforms and trends in the ophthalmology workforce and cataract surgery performance in Kazakhstan. Hum Resour Health. (2026) 24:8. doi: 10.1186/s12960-026-01047-1 PMC12888199 41530750

[23] Zhang F Wagner AK Ross-Degnan D. Simulation-based power calculation for designing interrupted time series analyses of health policy interventions. J Clin Epidemiol. (2011) 64:1252–61. doi: 10.1016/j.jclinepi.2011.02.007 21640554

[24] Muggeo V. Estimating regression models with unknown break-points. Stat Med. (2003) 22:3055–71. doi: 10.1002/sim.1545 12973787

[25] Ministry of Health of the Republic of Kazakhstan. Nurses' Day: on enhancing the role and status of mid-level specialists in Kazakhstan. gov.egov.kz (2025) Available online at: https://www.gov.kz/memleket/entities/dsm/press/news/details/992955 (Accessed May 6, 2026).

[26] Ballena-Caicedo J Vera-Ponce VJ. Trends in the hypertension care cascade under dual guideline criteria in Peru, 2014–2024: a decade of nationally representative surveys with joinpoint analysis and projections toward the WHO 2030 targets. Front Public Health (2026) 14:1830773. doi: 10.3389/fpubh.2026.1830773 PMC13272369 42317979

[27] Newey WK West KD. A simple, positive semi-definite, heteroskedasticity and autocorrelation consistent covariance matrix. Econometrica. (1987) 55:703–8. doi: 10.2307/1913610

[28] Andrews DWK. Heteroskedasticity and autocorrelation consistent covariance matrix estimation. Econometrica. (1991) 59:817–58. doi: 10.2307/2938229

[29] Hyndman RJ Khandakar Y. Automatic time series forecasting: the forecast package for R. J Stat Softw. (2008) 27:1–22. doi: 10.18637/jss.v027.i03

[30] Cabrera E Zabalegui A. Bologna process in European nursing education. Ten years later, lights and shadows. J Adv Nurs. (2021) 77:1102–4. doi: 10.1111/jan.14727 33314242

[31] Antão C Santos B Santos N Fernandes H Barroso B Mǎrginean CO . Nursing degree curriculum: differences and similarities between 15 European countries. Nurs Rep. (2025) 15:112. doi: 10.3390/nursrep15030112 40137684 PMC11946740

[32] Taneva D Paskaleva D Gyurova-Kancheva V. Nursing education in some European higher education area (EHEA) member countries: a comparative Analysis. Iran J Public Health. (2023) 52:1418–27. doi: 10.18502/ijph.v52i7.13243 37593510 PMC10430398

[33] Buchan J Catton H Shaffer F. Sustain and retain in 2022 and beyond: the global nursing workforce and the COVID-19 pandemic. Geneva: International Council of Nurses. (2022). Available online at: https://www.icn.ch/resources/publications-and-reports/sustain-and-retain-2022-and-beyond (Accessed May 5, 2026).

[34] Buchan J O'May F Dussault G. Nursing workforce policy and the economic crisis: a global overview. J Nurs Scholarship. (2013) 45:298–307. doi: 10.1111/jnu.12028 23656542

[35] Alameddine M Baumann A Laporte A Deber R A. narrative review on the effect of economic downturns on the nursing labour market: implications for policy and planning. Hum Resour Health. (2012) 10:23. doi: 10.1186/1478-4491-10-23 22905739 PMC3472285

[36] Kadyrova N Chanturidze T. “Economic crisis, health systems and health in Europe: country experience. Copenhagen: WHO Regional Office for Europe, European Observatory on Health Systems and Policies (2015). Chapter: Kazakhstan.,” *Economic crisis, health systems and health in Europe: Country experience [Internet]*. European Observatory on Health Systems and Policies (2015) Available online at: https://www.ncbi.nlm.nih.gov/books/NBK447870/ (Accessed September 3, 2026).

[37] Kulkayeva G. Medical migration in the republic of Kazakhstan: internal and external trends, factors, and possible solutions. J Health Dev. (2025) 60:jhd033. doi: 10.32921/2663-1776-2025-60-6-jhd033

[38] Sydykova B Smailova D Khismetova Z Brimzhanova M Baigozhina Z Hosseini H . Enhancing nursing documentation in Kazakhstan: assessing utilization and standardization for improving patient care. Front Public Health (2023) 11:1722480. doi: 10.3389/fpubh.2023.1267809 38074771 PMC10702541

[39] Kumarov K Baigozhina Z. Status and perspectives of nursing development in the Republic of Kazakhstan. J Health Dev. (2022) 2:23–32.

[40] WHO Regional Office for Europe. Strategic directions for nursing and midwifery in central Asia (2025–2030). (2025) Available online at: https://www.who.int/europe/publications/i/item/WHO-EURO-2025-12756-52530-81184 (Accessed April 30, 2026).

[41] OECD. Health at a Glance 2025: OECD Indicators. Health at a Glance (2025) 2025. doi: 10.1787/8f9e3f98-en

[42] Almazan J Demidenko A Kassimova G Majanova M Zhienbayeva B Asanova A . Analyzing the drivers of job satisfaction and nurse retention among Kazakh nurses in public clinical centers: key factors and insights. Nursing Forum. (2026) 2026:4032456. doi: 10.1155/nuf/4032456

[43] Lee Y Shin H-Y. Rushed health workforce reform in South Korea: a Kingdon's multiple streams framework analysis of the 2024 medical school quota expansion. Front Public Health (2025) 13:1673605. doi: 10.3389/fpubh.2025.1673605 41306869 PMC12644070

