# Supplementary material for: Nursing workforce trends in Kazakhstan, 2003–2024: an interrupted time series and joinpoint regression analysis in the context of educational reform
Source: Front Public Health. 2026 Sep 28;14:1913586. doi: 10.3389/fpubh.2026.1913586 (PMC13655116; doi:10.3389/fpubh.2026.1913586)
Supplement: Supplementary file 1 [file Data_Sheet_1.docx]

Supplementary Material

*Nursing Workforce Trends in Kazakhstan, 2003–2024: An Interrupted Time Series and Joinpoint Regression Analysis in the Context of Educational Reform*

**Supplementary Table S1. Timeline of nursing education reforms in Kazakhstan**

| **Year** | **Event** | **Role in analysis** |
| --- | --- | --- |
| 2007 | Bachelor of Nursing introduced at universities | Covariate (secondary analysis) |
| 2009 | Rural healthcare workforce incentive scheme |  |
| 2010 | Kazakhstan joined the Bologna Process |  |
| 2014 | Applied Bachelor programme initiated (MoH Order No. 417) | Primary intervention (policy initiation phase) |
| 2015 | Law on Education amended: applied bachelor and higher college defined |  |
| 2016 | Accelerated track for working nurses; Densaulyk programme; Roadmap adopted |  |
| 2017 | State educational standards (GOSO) formally approved (MoH Order No. 471) | Sensitivity analysis S6 (formal implementation phase) |
| 2018 | First Applied Bachelor graduates; expanded rollout |  |
| 2020 | Mandatory social health insurance (MSHI); COVID-19 pandemic |  |
| 2022 | Updated state education standards (MoH Order No. QR DSM-63) |  |

*MoH, Ministry of Health of the Republic of Kazakhstan; GOSO, State Compulsory Educational Standards; MSHI, Mandatory Social Health Insurance. The role-in-analysis column indicates how each reform point was used in the interrupted time series analysis. Source: legal information system Adilet (adilet.zan.kz).*

**Supplementary Table S2. Regional inequality in nursing workforce density in Kazakhstan, 2003 to 2024**

| **Year** | **Regions (n)** | **Mean** | **SD** | **CV (%)** | **Min** | **Max** | **Lowest region** | **Highest region** | **Max/min** |
| --- | --- | --- | --- | --- | --- | --- | --- | --- | --- |
| 2003 | 16 | 79.3 | 12.7 | 16.1 | 52.3 | 103.1 | Almaty region | Kyzylorda | 2.0 |
| 2004 | 16 | 80.2 | 12.4 | 15.5 | 52.3 | 104 | Almaty region | Kyzylorda | 2.0 |
| 2005 | 16 | 81.4 | 12.1 | 14.9 | 52.8 | 104.1 | Almaty region | Kyzylorda | 2.0 |
| 2006 | 16 | 84.3 | 12.8 | 15.2 | 58.3 | 118.2 | Almaty region | Kyzylorda | 2.0 |
| 2007 | 16 | 87.1 | 12.5 | 14.4 | 58.5 | 118.3 | Almaty region | Kyzylorda | 2.0 |
| 2008 | 16 | 87.7 | 13.7 | 15.7 | 52.6 | 119.4 | Almaty region | Kyzylorda | 2.3 |
| 2009 | 16 | 100.4 | 25.2 | 25.1 | 78.8 | 183.8 | Aktobe | East Kazakhstan | 2.3 |
| 2010 | 16 | 102.3 | 25.1 | 24.6 | 79.5 | 183.2 | Atyrau | East Kazakhstan | 2.3 |
| 2011 | 16 | 112.1 | 23.6 | 21 | 90.7 | 186.2 | Mangystau | East Kazakhstan | 2.1 |
| 2012 | 16 | 116.3 | 25.4 | 21.8 | 91.7 | 194.3 | Kostanay | East Kazakhstan | 2.1 |
| 2013 | 16 | 116 | 27.1 | 23.4 | 91.3 | 200.9 | Almaty city | East Kazakhstan | 2.2 |
| 2014 | 16 | 107 | 27.3 | 25.5 | 80.9 | 196.2 | Kostanay | East Kazakhstan | 2.4 |
| 2015 | 16 | 108.2 | 27.4 | 25.3 | 81.2 | 196.5 | Kostanay | East Kazakhstan | 2.4 |
| 2016 | 16 | 110 | 28.3 | 25.7 | 80.1 | 197.6 | Kostanay | East Kazakhstan | 2.5 |
| 2017 | 16 | 111.6 | 27.5 | 24.7 | 83.7 | 199.3 | Kostanay | East Kazakhstan | 2.4 |
| 2018 | 17 | 107.1 | 26.2 | 24.4 | 81.4 | 198.4 | Kostanay | East Kazakhstan | 2.4 |
| 2019 | 17 | 107.9 | 27.9 | 25.9 | 81.2 | 204.6 | Kostanay | East Kazakhstan | 2.5 |
| 2020 | 17 | 110.1 | 30 | 27.2 | 82.3 | 216.3 | Kostanay | East Kazakhstan | 2.6 |
| 2021 | 17 | 110.1 | 29.1 | 26.5 | 81 | 211.5 | Kostanay | East Kazakhstan | 2.6 |
| 2022 | 20 | 99.2 | 12.7 | 12.8 | 72.3 | 131.8 | Almaty region | Kyzylorda | 1.8 |
| 2023 | 20 | 98.1 | 13.1 | 13.3 | 70.6 | 134.8 | Almaty region | Kyzylorda | 1.9 |
| 2024 | 20 | 97.4 | 13.3 | 13.6 | 72 | 134.8 | Almaty region | Kyzylorda | 1.9 |

*Nursing density expressed as mid-level medical personnel per 10,000 population. SD, standard deviation; CV, coefficient of variation; Max/min, ratio of the highest to the lowest regional density. The increase in CV from 2009 coincides with population rebasing following administrative boundary changes; the decrease from 2022 coincides with the creation of three new regions (Abay, Zhetysu, Ulytau). The number of regions changed from 16 (2003–2017) to 17 (2018–2021, following creation of Turkestan region and Shymkent city) to 20 (2022–2024). Source: Bureau of National Statistics of Kazakhstan.*

**Supplementary Table S3. Full annual ARIMA forecast of nursing workforce density in Kazakhstan to 2035**

| **Year** | **Point forecast** | **80% prediction interval** | **95% prediction interval** |
| --- | --- | --- | --- |
| 2025 | 95.9 | 92.9 to 98.8 | 91.3 to 100.4 |
| 2026 | 96.6 | 91.2 to 102.1 | 88.3 to 105.0 |
| 2027 | 97.9 | 90.4 to 105.3 | 86.4 to 109.3 |
| 2028 | 99.3 | 90.3 to 108.3 | 85.6 to 113.1 |
| 2029 | 101.0 | 90.8 to 111.1 | 85.5 to 116.4 |
| 2030 | 102.7 | 91.8 to 113.6 | 86.0 to 119.3 |
| 2031 | 104.4 | 93.0 to 115.8 | 87.0 to 121.8 |
| 2032 | 106.1 | 94.4 to 117.8 | 88.2 to 124.0 |
| 2033 | 107.8 | 95.9 to 119.6 | 89.6 to 125.9 |
| 2034 | 109.4 | 97.4 to 121.4 | 91.1 to 127.7 |
| 2035 | 111.0 | 98.9 to 123.0 | 92.6 to 129.4 |

*ARIMA(2,0,0) model with intervention and time-after-intervention as external regressors. Values are nursing and midwifery personnel per 10,000 population. The forecast assumes continuation of current policy conditions and should be regarded as indicative rather than precise given the limited series length. Selected years from this table are presented in Table 5 of the main text.*

**Supplementary Figure S1.** Autocorrelation (left) and partial autocorrelation (right) functions of the normalised residuals from the primary GLS-AR(2) model. Dashed lines indicate 95% confidence bounds. No coefficient exceeds the bounds, and Ljung-Box tests on the same residuals were non-significant at lags 1, 2, 3 and 5 (p = 0.97, 0.99, 0.38 and 0.61), indicating that the second-order autoregressive structure adequately captures the serial dependence in the series.
